# Hand Osteoarthritis: Molecular Mechanisms, Randomized Controlled Trials, and the Future of Targeted Treatment

**DOI:** 10.3390/ijms26104537

**Published:** 2025-05-09

**Authors:** Yemisi D. Joseph, Amy L. Ladd, Nidhi Bhutani

**Affiliations:** 1 Stanford University School of Medicine, Stanford University, Palo Alto, CA 94305, USA; yemgrey@stanford.edu; 2 Department of Orthopaedic Surgery, Stanford University, Redwood City, CA 94063, USA; alad@stanford.edu

**Keywords:** hand osteoarthritis, thumb CMC osteoarthritis, genetic variants, inflammatory cytokines, epigenetics, gut–joint axis, DNA methylation, microbiome dysbiosis, adipokines, randomized controlled trials

## Abstract

Hand osteoarthritis (OA) is a prevalent and disabling condition, yet its pathogenesis remains less studied than OA in large weight-bearing joints. Emerging genetic, epigenetic, and microbiome research suggests that hand OA might be biologically distinct, involving joint-specific pathways not shared by knee or hip OA. This review integrates genome-wide association studies specific to hand OA, highlighting key molecular contributors such as inflammatory cytokines. These genetic insights, together with emerging data on epigenetic alterations and gut microbial dysbiosis, point to broader systemic and regulatory influences on hand OA onset and progression. We also assess pharmacologic interventions tested in randomized controlled trials that have attempted to target these pathways. While agents such as TNF and IL-6 inhibitors, hydroxychloroquine, and corticosteroids have shown limited success, emerging evidence supports the potential of methotrexate in synovitis-positive general hand OA, platelet-rich plasma in thumb carpometacarpal (CMC) OA, and prolotherapy in interphalangeal (IP) OA. These findings illustrate the persistent gap between mechanistic understanding and therapeutic success. Future work must prioritize multifactorial strategies for addressing pain and translational frameworks that link molecular mechanisms to treatment response. In summary, this review offers an update on hand OA and identifies key opportunities for more targeted and effective therapy.

## 1. Introduction

Osteoarthritis (OA) is a globally prevalent, progressive joint disorder characterized by the deterioration of cartilage and irreversible bone damage, leading to pain, stiffness, and reduced mobility [1,2]. It impairs patients’ quality of life, causes disability, and imposes significant healthcare costs [3,4]. The prevalence of hand OA in the general population is estimated to be approximately 12%, primarily affecting the interphalangeal (IP) joints—which include the distal (DIP) and proximal (PIP) joints—as well as the carpometacarpal (CMC) joint of the thumb [5]. Notably, thumb CMC OA is characterized by a disproportionately higher functional burden, likely due to the central role of the thumb in critical hand functions such as precision grip, pinching, and object manipulation [6,7].

Although hand OA encompasses a spectrum of phenotypes—including erosive disease and inflammatory arthritis such as rheumatoid arthritis (RA)—this review focuses specifically on primary, non-erosive, non-autoimmune hand OA, which remains the most prevalent and clinically relevant subtype. Erosive hand OA is associated with more aggressive clinical progression and distinct inflammatory and genetic features, while RA of the hand reflects a fundamentally different autoimmune pathology. Given these differences in pathogenesis and management, a dedicated review of those subtypes is beyond the scope of this article.

### 1.1. Epidemiology of Hand OA

The global incidence of hand OA increased by more than 80% from 1990 to 2019, despite a slight decline in the age-standardized incidence rate [8]. Women exhibited a higher incidence and disability burden across all age groups, with a peak between ages 50 and 54 [8]. Although disease rates varied by region, the overall global rise was largely driven by aging populations and increased life expectancy [8]. Prevalence estimates vary depending on the definition used (clinical vs. radiographic) and the population studied. Radiographic hand OA is present in approximately 40–60% of adults over age 55, while symptomatic hand OA affects about 13–26% of individuals in the same age group [5,9,10]. The condition disproportionately affects women, particularly postmenopausal women, who experience higher rates of symptomatic disease, polyarticular involvement, and more rapid structural progression compared to men [5,11]. Age-adjusted prevalence studies confirm these trends and highlight sex-specific patterns of joint involvement: women more frequently exhibit DIP and PIP joint disease, while men show relatively greater involvement of the metacarpophalangeal (MCP) joints [5].

Risk factors for hand OA include increasing age, female sex, family history of OA, repetitive hand use, obesity, metabolic syndrome, and systemic low-grade inflammation [5,12]. Ethnic disparities have also been observed, with lower prevalence and incidence reported among Black populations compared to White populations [5]. Emerging data also point to links between hand OA and cardiovascular disease, diabetes, and adipokines such as leptin—further supporting a systemic dimension to the disease’s pathophysiology [6].

### 1.2. Treatment of Hand OA

The management of hand OA—including both IP and thumb CMC involvement—is typically multimodal and tailored to disease severity, joint involvement, and patient-specific goals. Foundational nonpharmacologic strategies such as patient education, joint protection techniques, hand exercises, and splinting are consistently emphasized across international guidelines, including those from the American College of Rheumatology, EULAR, and NICE [12,13,14]. Among these, splinting plays a particularly central role in thumb CMC OA, where it can reduce pain and preserve function essential for precision grip and daily hand use [15,16,17].

Topical NSAIDs are recommended as first-line pharmacologic therapy due to their efficacy and safety, especially in older adults [18,19]. Oral NSAIDs, while effective, are generally reserved for cases refractory to other measures and require careful monitoring due to systemic toxicity risks [20,21].

Intra-articular corticosteroids may offer short-term pain relief in patients with IP joint inflammation but appear to be less effective for thumb CMC OA [22,23]. Surgical options—including trapeziectomy, arthrodesis, or joint arthroplasty—are considered for patients with persistent symptoms and structural joint damage unresponsive to non-surgical management [24,25,26,27]. Additional topical agents, nutraceuticals, and systemic therapies such as capsaicin, diacerein, hydroxychloroquine, and methotrexate have been evaluated, but most lack robust evidence and are not routinely recommended due to modest efficacy or safety concerns [28,29,30]. Despite the breadth of available treatment options, no therapy has yet demonstrated structural disease-modifying effects in hand OA—highlighting the urgent need to develop treatments that directly target the pathogenic mechanisms reviewed in the following sections.

### 1.3. Mechanisms Underlying the Molecular Pathogenesis of Hand OA

Despite the high prevalence of hand OA and its burden, its molecular mechanisms remain less well understood compared to those of knee and hip OA [6]. Recent genome-wide association studies (GWASs) have revealed that while OA across different joints shares several common pathways, there also exist unique joint-specific genetic and molecular pathways at play as well [31,32,33]. A comprehensive GWAS meta-analysis involving over 826,000 individuals from nine populations highlighted genetic loci specifically associated with hand OA and distinct from those linked to weight-bearing joints such as the knee and hip [31]. The study identified several risk variants unique to non-weight-bearing joints, including hand and thumb OA, suggesting a distinct genetic architecture underpinning OA in different joints. Identification of variants uniquely associated with hand OA also supports the notion that hand OA’s molecular mechanisms may differ from those that drive OA in other joints [31].

The inflammatory processes involved in hand OA pathogenesis are increasingly characterized and genetic variants in these inflammatory factors have been identified. Inflammation plays a critical role in cartilage degradation, and pro-inflammatory cytokines such as tumor necrosis factor alpha (TNF-α), interleukin-1 beta (IL-1β), and interleukin-6 (IL-6) have been implicated in the progression of OA in multiple joints, including the hand [34,35,36,37]. These cytokines contribute to the upregulation of matrix metalloproteinases (MMPs), which accelerate the breakdown of cartilage extracellular matrix components, further exacerbating joint damage [38,39]. Understanding the molecular interplay between these cytokines and their impact on chondrocyte function and synovial inflammation in hand OA is crucial for identifying therapeutic targets. In addition to genetic predisposition and inflammation, epigenetic regulation has emerged as another major factor in OA pathogenesis [40,41,42,43,44]. This emerging research into the role of epigenetic modifications, particularly DNA methylation, in modulating gene expression in OA-affected joints may explain why certain joints, such as the hand, are more susceptible to OA progression despite shared genetic predispositions.

Given the complexity and joint-specific nature of hand OA, this review aims to serve as a reference of our current and unfolding understanding of the molecular pathogenesis specific to hand OA, as well as the pharmacologic therapies that have sought to target these mechanisms and evaluate their efficacy via randomized controlled trials (RCTs).

In summary, we highlight the genetic factors associated with hand OA with a particular focus on those involved in inflammatory pathways. In addition, we highlight the emerging roles of epigenetics and the gut–joint axis in the pathogenesis of hand OA and discuss how these mechanistic insights have informed, and in some cases diverged from, clinical outcomes observed in RCTs.

## 2. Methods

To critically examine and consolidate our current knowledge on the underlying pathogenesis of hand OA, we conducted a narrative review focused on—but not limited to—primary, non-erosive, and non-autoimmune forms of the disease. To explore the molecular and genetic mechanisms of hand OA, we developed a strategy to identify relevant GWASs, preclinical models, and translational human studies. We searched PubMed, Embase, and Google Scholar for articles published between January 2000 and February 2025. Search terms were structured as Boolean intersections between anatomical descriptors—*hand osteoarthritis, thumb carpometacarpal OA, first CMC joint, distal interphalangeal (DIP), proximal interphalangeal (PIP)*, and *interphalangeal (IP)*—and broad mechanistic themes, including *genetics, GWAS, cartilage degradation, synovium, inflammation, chondrocyte, epigenetics, DNA methylation, microRNAs, cytokines, gut microbiome,* and *adipokines.*

This strategy yielded 894 articles, which were screened by title and abstract for relevance. Full-text review was conducted for studies addressing core molecular or joint-specific mechanisms in hand OA. Although inclusion was not strictly limited to primary hand OA, we excluded studies focused exclusively on erosive OA or rheumatoid arthritis due to their distinct autoimmune or inflammatory pathogenesis. Additionally, studies centered on other OA joints such as knee or hip OA were excluded due to their lack of joint-specific relevance to the molecular and clinical features unique to hand OA.

Once a genetic variant, transcriptomic signal, or pathway was implicated in hand OA through GWAS, differential expression, or protein analysis in human hand tissue, we sought to contextualize these findings using functional studies—including in vitro systems and animal models—to clarify the biological role of the implicated target. While many of these mechanistic studies were not specific to hand OA, we incorporated them where appropriate to hypothesize plausible cellular mechanisms while clearly noting when extrapolation from other joints or models was required.

When the specific joint or hand OA subtype (e.g., thumb CMC, DIP, or PIP) was not explicitly stated in a study, we refer to it as “hand OA” in this review. Where the original publication identified the precise joint(s) involved, we retained that level of specificity. This approach allows for consistent interpretation of findings across a heterogeneous body of literature while preserving alignment with how disease involvement was characterized in each study. It also reflects a practical necessity, as many large-scale studies and trials on hand OA often report composite outcomes or aggregate diagnoses without specifying joint-level detail.

## 3. Tumor Necrosis Factor Alpha (TNFα) and the Interleukins

### 3.1. Tumor Necrosis Factor Alpha (TNFα)

**Figure 1:**
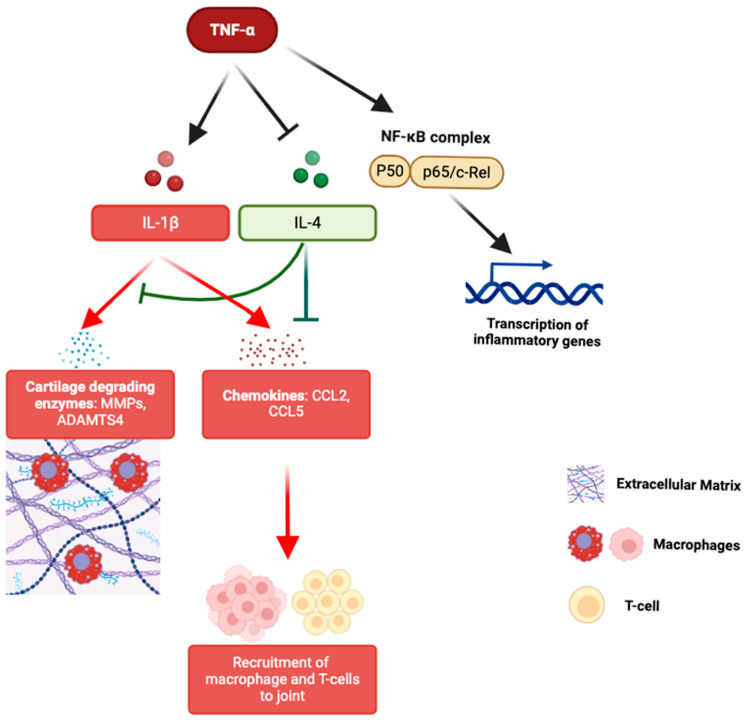
TNF-α-driven inflammatory pathways in joint degradation. TNF-α activates IL-1β signaling while inhibiting the chondroprotective IL-4 pathway, leading to cartilage degradation (MMPs, ADAMTS4), chemokine production (CCL2, CCL5), immune cell recruitment, and NF-κB-mediated transcription of inflammatory genes.

TNFα is a pro-inflammatory cytokine that plays a central role in the inflammatory processes associated with OA [34,35,36]. Significant associations between two TNFα polymorphisms (*rs1799964* and *rs1800630*) and an increased risk of hand OA have been identified [37]. Additionally, the G-A-G haplotype of TNFα was linked to a higher likelihood of developing hand OA. Interactions between TNFα polymorphisms and variants in IL-4R and IL-10 genes have also been observed, suggesting a gene-interaction network effect. Thus, TNFα polymorphisms may function singularly or together with other cytokines such as IL-1β, IL-4, IL-6, and IL-10 to initiate and sustain inflammation (Figure 1). Furthermore, the observed combined effect of TNFα, IL-1β, and IL-6 polymorphisms was larger but less than additive, indicating a potential synergistic interaction among these cytokines in promoting inflammation and joint damage in hand OA.

Recent single-cell RNA sequencing (scRNA-seq) studies have found that specific chondrocyte subpopulations, such as fibrocartilage chondrocytes (FCs) and inflammatory chondrocytes (InflamCs), are enriched in hand OA [45]. These subpopulations were enriched in pathways related to reactive oxygen species, TNFα signaling via nuclear factor kappa B (NF-κB), and various interleukin-mediated pathways. These findings suggest that TNFα and its gene network can modulate the inflammatory milieu of hand OA via these specialized chondrocyte populations, thereby contributing to the disease’s pathogenesis (Figure 1).

### 3.2. Interleukin-1 (IL-1)

Multiple studies have implicated IL-1 in the pathogenesis of OA across multiple joints [39,46,47]. In vitro, IL-1 promotes cartilage degradation via various mechanisms, including acting synergistically with TNFα, inducing major cartilage degradation enzymes such as MMPs and ADAMTS4, driving the expression of chemokines such as CCL2 and CCL5, which recruit inflammatory cells such as macrophages and T cells, thereby amplifying synovial inflammation (Figure 1) [48,49]. *IL1B rs1143633* minor allele, when present in a homozygous state, has been identified to be significantly associated with hand OA (OR 3.4, *p* = 0.006) [47]. Carriers of the *IL1B rs1143634* minor allele have also been found to have an increased DIP OA risk (OR 1.6; 95% CI 1.08–2.26) compared to noncarriers [50]. The association was stronger in dentists, a profession characterized by repetitive manual tasks and high cumulative hand loading, implying an interaction between repetitive occupational mechanical stress and genetic susceptibility. Additionally, the extended *IL1A-IL1B-IL1RN* haplotype containing this allele is also associated with an increased risk of hand OA, although the association is stronger with hip OA. This distinction highlights the complexity of OA genetics and the joint-specific variability in the disease pathophysiology. Taken together, these findings suggest that specific *IL-1* haplotypes may predispose DIP joints to OA by modulating inflammatory responses.

### 3.3. Interleukin-4 and Its Receptor (IL-4/IL-4R)

The IL-4/IL-4R pathway is chondroprotective, preventing the development of generalized OA [51,52,53]. IL-4 decreases IL-1β-induced protein expression of MMP-13, a major degrading enzyme of extracellular matrix (ECM) proteins in cartilage such as collagen and aggrecan [54,55,56]. IL-4 also decreases normal T expressed and secreted (RANTES)/CC ligand 5 (CCL5), a chemokine that induces further MMPs activity and attracts immune cells into the affected joint (Figure 1) [54,57]. Two SNPs (*rs1805013* and *rs1805015*) in the *IL-4R* gene were found to be linked to hand OA in a case–control association study involving 403 patients with hand OA and 322 healthy controls [58]. The significance of these associations was more pronounced for non-erosive primary hand OA, suggesting that these genetic variants may impair IL-4-mediated chondroprotection.

### 3.4. Interleukin-6 (IL-6)

IL-6 is a pro-inflammatory cytokine known to be involved in the regulation of immune responses and inflammation. Its role in generalized OA has been highlighted through its ability to promote cartilage degradation [59,60]. *rs1800795* and *rs1800797* polymorphisms in the IL-6 gene, which correlate with higher levels of IL-6, have been associated with an increased risk and severity of hand OA [61]. IL-6 promoter variants (*G-597A* and *G-174C*) have also been implicated in symptomatic DIP OA [62]. Subjects with symptomatic DIP OA more commonly possessed G alleles at the *G-597A* and *G-174C* locations than those without the disorder. Having at least one G allele was associated with a higher likelihood of this disease. Moreover, those with a haplotype that includes the G allele in promoter SNPs were seen to have a quadrupled risk of developing symptomatic DIP OA. Carrying the *G-G diplotype* has been linked to a higher risk of both symmetrical and symptomatic DIP OA. The study highlighted that the G alleles at these variable regulatory regions for IL-6 are associated with increased transcription and secretion of IL-6, contributing to the inflammatory processes underlying OA. IL-6 promoter and gene polymorphisms could therefore be further investigated as potential biomarkers both for predicting the risk and progression of DIP hand OA.

## 4. The Adipokines

Adipocytes secrete several adipokines, including adiponectin, leptin, and resistin, which have both pro- and anti-inflammatory roles [63,64]. For example, lower adiponectin levels in serum were found to correlate significantly with the advancement of radiographic hand OA and lower baseline adiponectin levels were found to inversely correlate with the worsening of radiographic hand OA [65]. These findings suggest that adiponectin, a protein hormone involved in regulating glucose levels and fatty acid breakdown, plays a protective role in hand OA, and its reduced levels may facilitate disease progression. Recent studies have identified two single-nucleotide polymorphisms (SNPs) in the adipokines chemerin and resistin—namely, *rs4721* in the *RARRES2* (*chemerin*) gene and *rs3745368* in the *RETN* (*resistin*) gene—that are associated with an increased risk of hand OA [66]. Specifically, the G allele of *rs4721* and the A allele of *rs3745368* are linked to a higher risk of hand OA, with odds ratios (ORs) of 1.25 and 1.33, respectively [66]. Furthermore, while the G allele in *rs4721* was associated with higher pain analog scales (PASs) in hand OA patients, indicating a link between this genetic variant and the severity of pain experienced, the A allele of *rs3745368* was associated with more severe Kellgren–Lawrence (KL) grades in hand OA patients, suggesting a role in disease progression.

**Figure 2:**
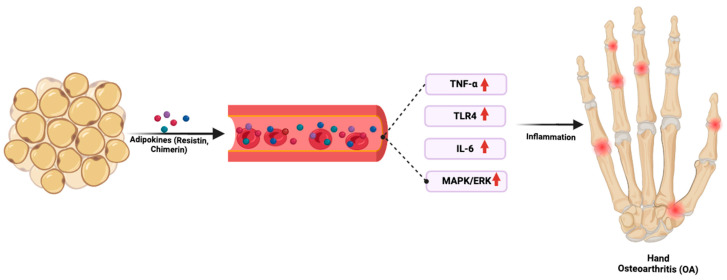
Adipokine-driven inflammation linking obesity to hand OA. Adipokines such as resistin and chemerin from adipose tissue trigger systemic inflammation via upregulation of TNF-α, TLR4, IL-6, and MAPK/ERK pathways, promoting joint inflammation and the development of hand OA.

These results are consistent with the prior literature, which has implicated both resistin and chemerin in the inflammatory response involved in the pathophysiology of various diseases [67,68]. For example, resistin, which is elevated in hand OA, is known to interact with TNF and IL-6, which are key pro-inflammatory cytokines (Figure 2) [69,70]. Similarly, chemerin has been shown to induce several pro-inflammatory cytokines through the activation of toll-like receptor 4 (TLR4) and mitogen-activated protein kinase / extracellular signal-regulated kinase (MAPK/ERK) pathways [71,72]. A study examining adipokine gene associations with radiographic hand OA in Finnish women found weak associations between variations in leptin receptor (*LEPR*), *RARRES2*, and *RETN* genes and hand OA [73]. However, the associations were modified by BMI, suggesting that an individual’s adiposity may influence the impact of these genetic variations. In conclusion, adipokine dysregulation may drive chronic low-grade inflammation and systemic metabolic dysfunction, contributing to hand OA pathogenesis.

## 5. Other Pathways and Genes Implicated in Hand OA

### 5.1. Transforming Growth Factor Alpha (TGFα)

The variant *rs3771501* in *TGFA* was implicated in hand OA in a recent large-scale GWAS, suggesting a potential joint-specific role of TGFα in hand OA pathogenesis [31]. TGFα signals through the epidermal growth factor receptor (EGFR) to regulate processes such as cell proliferation, differentiation, and response to mechanical stress and is known to play a central role in generalized OA [74,75,76]. Elevated levels of TGFα have been detected in the synovial membrane and synovial fluid of patients with knee OA, as well as in surgically induced OA models (not specific to the hand), with expression increasing fourfold in chondrocytes after joint injury [77,78]. While these data derive from non-hand OA studies, they provide insight into possible mechanisms through which TGFα may contribute to synovial inflammation and cartilage degradation in hand OA.

TGFα’s effect on chondrocytes is highly context-dependent and can be either beneficial or pathological. In vitro experiments using a cell-stretching system revealed that recombinant TGFα reduced chondrocyte apoptosis and enhanced autophagy when administered prior to mechanical stress [79]. This effect was accompanied by decreased expression of MMP-13 and increased levels of anabolic markers, such as aggrecan (ACAN), suggesting that TGFα can protect chondrocytes under mechanical load. However, the protective effects of TGFα were reversed in animal models when subjected to increased physical activity, leading to more pronounced cartilage degeneration in TGFα KO mice. While inhibition of TGFα or its receptor EGFR shows potential in reducing the catabolic activity of chondrocytes and slowing OA progression, the context-specific effects of this pathway—particularly in relation to mechanical stress—must be carefully considered before any therapeutic strategies can be considered. Future studies are needed to clarify the mechanisms by which TGFα contributes to hand OA, particularly under varying mechanical conditions.

### 5.2. Matrix Gla Protein (MGP)

MGP, a vitamin K-dependent ECM protein, plays a critical role in preventing ectopic calcification in cartilage and bone [80]. Genetic variation in *MGP* has been implicated in hand OA; a recent GWAS identified that the hand OA risk allele was associated with lower MGP RNA expression in cartilage, suggesting reduced function of this mineralization inhibitor [80]. Given that the carboxylation and activation of MGP require adequate vitamin K, this raises the possibility that vitamin K insufficiency may heighten susceptibility to cartilage degeneration and joint damage in hand OA [81].

Observational and interventional studies have explored this hypothesis more directly. In the Framingham Offspring Study, lower plasma phylloquinone levels—a marker of vitamin K status—were significantly associated with increased prevalence of radiographic hand OA, large osteophytes, and joint space narrowing [82]. A follow-up randomized controlled trial of vitamin K supplementation in older adults found no overall effect on hand OA outcomes. However, a subgroup analysis revealed that participants with low baseline vitamin K levels who achieved sufficient levels post-treatment had a 47% lower prevalence of joint space narrowing at follow-up, suggesting that vitamin K insufficiency may contribute to OA progression in susceptible individuals [83].

Together, these findings suggest that both genetic and nutritional modulation of MGP activity may influence the structural integrity of hand joints. Further targeted studies are warranted to clarify whether restoring vitamin K sufficiency can modify hand OA progression in high-risk subgroups.

### 5.3. Nuclear Receptor Subfamily 3 Group C Member 1 (NR3C1)

Another variant associated with hand OA is *rs10062749* in the *NR3C1* gene, which encodes the glucocorticoid receptor, a key mediator of the body’s response to glucocorticoids in inflammatory reactions, cell growth, and tissue differentiation [31]. In hand OA, this variant may influence disease progression by modulating sensitivity to glucocorticoids, which have well-established anti-inflammatory properties. For example, polymorphisms such as *ER22/23EK* and *9β* are associated with increased risk of RA, while other variants, like *Asn363Ser* and *BclI*, enhance glucocorticoid sensitivity, leading to a range of effects from metabolic disturbances to altered immune responses [84,85].

The critical role of *NR3C1* in hand OA is further supported by its influence on other glucocorticoid-responsive genes, such as *DUSP1*, *ANXA1*, and *GILZ*. These genes mediate anti-inflammatory effects, with DUSP1 inhibiting MAP kinase signaling and ANXA1 suppressing leukocyte migration [86]. However, the role of GILZ may be more complex, as it has been implicated in both anti-inflammatory processes and OA pathogenesis through its regulation of leptin expression in synovial fibroblasts, which has been linked to cartilage degeneration [86]. Although these findings are derived from models not exclusive to hand OA, they suggest potential relevance to cartilage degeneration in hand joints. Nevertheless, the effects of glucocorticoids on cartilage homeostasis appear to be dose-dependent and context-specific. While low-dose glucocorticoids have protective effects, high-dose or long-term administration can lead to cartilage damage and osteonecrosis [87]. In hand OA, this suggests that careful modulation of glucocorticoid signaling, potentially through targeting *NR3C1* variants, may represent a therapeutic strategy that balances inflammation control with cartilage preservation.

### 5.4. TEA Domain Transcription Factor 1 (TEAD1)

While there have not been any mechanistic studies linking TEAD1 to hand OA, a GWAS study identified the *TEAD* variant, *rs3993110*, as being significantly associated with hand OA, suggesting a potential role in disease susceptibility [31]. TEAD1 is primarily known for its interaction with YAP (Yes-associated protein) and TAZ (Transcriptional coactivator with PDZ-binding motif), two critical effectors of the Hippo signaling pathway involved in cartilage homeostasis and mechanotransduction [88,89,90]. Through its interaction with YAP/TAZ, TEAD1 may act as a mediator of hand OA pathogenesis.

**Figure 3:**
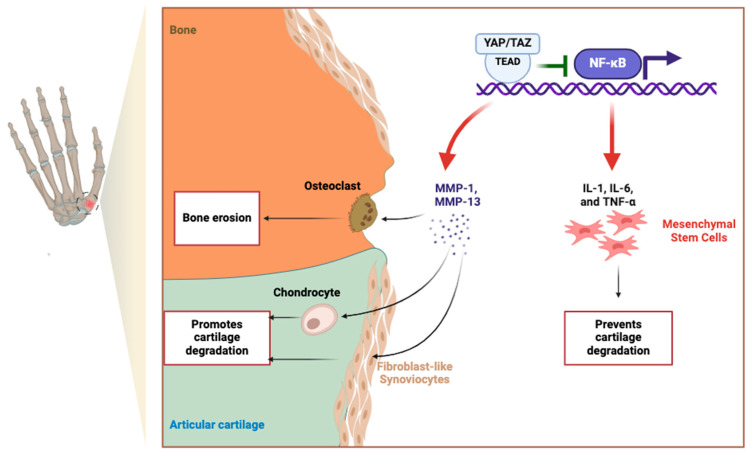
Dual roles of YAP/TAZ in fibroblast-like synoviocytes (FLSs) and mesenchymal stem cells (MSCs) in cartilage regulation. In FLSs, YAP/TAZ activation promotes MMP-1 and MMP-13 expression in response to TNF-α and IL-1β, driving ECM degradation. Conversely, in MSCs, YAP/TAZ–TEAD signaling enhances chondrogenesis by counteracting NF-κB signaling, thereby supporting cartilage repair and regeneration.

In cartilage biology, YAP/TAZ activity has been shown to exert dual effects, promoting either chondrocyte survival and repair or contributing to catabolic degradation, depending on cellular context and upstream stimuli [91,92]. In fibroblast-like synoviocytes (FLSs) derived from osteoarthritic joints not specific to hand OA, nuclear accumulation of YAP—especially when stimulated by TNF-α and IL-1β—leads to increased expression of matrix MMP-1 and MMP-13, thereby promoting extracellular matrix (ECM) breakdown (Figure 3) [91]. Conversely, in mesenchymal stem cells (MSCs), YAP/TAZ signaling through TEAD supports chondrogenic differentiation and represses NF-κB-mediated inflammation, thereby preserving ECM integrity and reducing MMP and ADAMTS expression (Figure 3) [91].

Beyond biochemical signaling, recent advances in mechanotransduction—the process by which cells sense and respond to mechanical cues—have demonstrated that YAP/TAZ activation is critically modulated by extracellular matrix stiffness, topographic fiber alignment, and cytoskeletal tension [90,93,94]. In engineered fibrous microenvironments, matrix stiffness and fiber orientation synergistically regulate YAP nuclear localization and transcriptional output, influencing both stem cell fate and inflammatory signaling [94]. This mechanosensitive behavior may be particularly relevant in hand OA, where altered joint loading and ECM disorganization could aberrantly activate YAP/TAZ, contributing to pathological tissue remodeling and impaired repair capacity [93]. Studies using human cartilage models have further shown that mechanical context alone—independent of biochemical signals—can reprogram chondrocyte behavior toward either a catabolic or reparative phenotype [95,96,97,98]. Together, these insights suggest that dysregulated mechanotransduction may be an underrecognized driver of hand OA pathogenesis and that the YAP/TAZ–TEAD1 axis warrants further investigation as a potential therapeutic target.

## 6. Thumb CMC OA: A Unique Subset of Hand OA

Thumb CMC OA is a prominent subtype of hand OA [99,100]. Its prevalence increases significantly with age, rising to 85% among individuals between 71 and 80 years of age [101]. As individuals increase in age, the prevalence becomes nearly universal, affecting 100% of women over 90 and 93% of men over 80 [101]. Furthermore, post-menopausal women with thumb CMC OA have greater odds of disease progression compared to their male counterparts, likely due to the impact of hormonal changes and biomechanical stressors unique to women [102]. One suspected reason for the high prevalence of thumb CMC OA is the unique mechanical demands of the thumb, which requires movements across multiple planes—such as flexion, extension, abduction, and opposition—making the CMC joint more susceptible to OA than other hand joints [103,104].

While thumb CMC OA shares common inflammatory and molecular pathways with generalized hand OA, additional and unique factors are involved in the pathogenesis of thumb CMC OA. These distinct mechanistic pathways merit specific attention, as they provide critical insights into why the thumb CMC joint is particularly vulnerable to OA compared to other hand joints. Thus, the following section will explore factors that are specific to thumb CMC OA, expanding upon the broader inflammatory mechanisms discussed earlier in the context of generalized hand OA.

The following sections will discuss the current understanding of the mechanistic pathways implicated in the pathogenesis and progression of thumb CMC OA, which may also help explain the observed sex differences in the disease.

### 6.1. Interleukin-7 (IL-7)

IL-7 is known to induce MMP13 expression and promote inflammatory T-cell activation, leading to increased secretion of pro-inflammatory cytokines that contribute to bone and cartilage degradation [105,106,107,108]. A recent study identified IL-7 as a potential biomarker for stratifying disease severity in thumb CMC OA, with higher circulating levels associated with a reduced likelihood of requiring surgical intervention [109]. These findings suggest a possible protective role for IL-7 in thumb CMC OA progression. However, elevated IL-7 levels have also been observed in older patients with OA compared to younger individuals [110,111,112], raising the possibility that age-related immunological changes—rather than disease activity alone—may contribute to these findings. Thus, further studies are needed to disentangle the IL-7-specific effects from age-related immune remodeling and to clarify whether IL-7 serves as a true disease-modifying factor or an age-associated phenomenon.

### 6.2. WNT9A (Wnt Family Member 9A)

*WNT9A* plays a role in chondrogenesis and maintaining joint integrity via SOX9, a transcription factor in chondrocyte differentiation and cartilage formation [113,114]. *rs1158850*, a variant within the regulatory region of the *WNT9A* gene, increases or decreases *WNT9A* expression via interactions between RAD21 cohesin complex and CTCF, forming a chromatin loop that regulates *WNT9A* gene expression levels [113]. The dysregulation of *WNT9A* expression leads to a decrease in SOX9 levels, subsequently impairing the synthesis of essential cartilage components like collagen type II. This, in turn, contributes to the pathogenesis and severity of thumb CMC OA by disrupting the balance of cartilage matrix production and degradation [114].

Supporting evidence from a study within the Chinese population further solidifies *WNT9A*’s association with thumb CMC OA, suggesting that the risk allele of *rs11588850* may not only elevate OA susceptibility through *WNT9A* upregulation but may also increase disease severity in patients with these variants [115]. However, the precise mechanism by which WNT signaling exacerbates thumb CMC OA, particularly in light of paradoxical findings that WNT pathway inhibition also aggravates disease phenotype, remains unresolved and requires further investigation [116,117].

### 6.3. ALDH1A2 (Aldehyde Dehydrogenase 1 Family, Member A2) and Retinoic Acid Metabolism

A population study from Iceland identified the *ALDH1A2* gene, which encodes retinaldehyde dehydrogenase 2 (RALDH2) as a potential molecular marker of thumb CMC OA [118]. RALDH2 is a key enzyme in the biosynthesis of all-trans retinoic acid (atRA) from retinaldehyde, an active metabolite of vitamin A known to play a critical role in the maintenance of cartilage and bone health [119,120,121]. Risk alleles in *ALDH1A2* that reduce atRA levels have been associated with increased cartilage inflammation, thus potentially influencing the initiation and progression of thumb CMC OA [118]. In addition, decreased retinoic acid production also likely compromises cartilage repair, contributing to the degenerative process characteristic of thumb CMC OA [118].

A recent study in the United Kingdom (UK) has further supported this association between the *ALDH1A2* gene and thumb CMC OA. In this study, variants of *ALDH1A2*, linked to the decreased synthesis of atRA, were identified in patients with severe hand OA [122]. These risk alleles were correlated with cartilage damage and inflammation, reinforcing the findings from the Icelandic population and extending them into a UK cohort. In addition, the UK study advanced our understanding of atRA’s anti-inflammatory properties in thumb CMC OA by demonstrating that the administration of a retinoic acid metabolism blocking agent (RAMBA), specifically talarozole, mitigated cartilage injury and inflammation by preserving atRA levels. This study corroborated the prior findings about *ALDH1A2* and further highlighted the potential of RAMBAs as a new class of disease-modifying drugs for thumb CMC OA treatment.

### 6.4. MATN3 (Matrilin-3)

Matrilin-3, an extracellular matrix protein encoded by the *MATN3* gene, plays a vital role in maintaining the structural and functional integrity of cartilage. *MATN3* polymorphisms, such as *p.T303M*, have been correlated with an increased risk of developing thumb CMC OA [123,124,125]. This association has been independently replicated in Icelandic and German population studies [123,125]. However, the role of *MATN3* polymorphisms in heterogeneous populations outside of these European groups remains unclear.

As an adaptor protein, matrilin-3 facilitates the interaction between collagen fibrils and aggrecan, among other cartilage constituents [126,127]. While these interactions have not been evaluated specifically in hand OA tissue, broader studies suggest that matrilin-3 also modulates chondrocyte metabolism and regulates the synthesis of ECM proteins [128]. The *p.T303M* polymorphism may alter the structure or function of matrilin-3, disrupting its role in ECM integration—an effect supported by in vivo findings in matrilin-deficient mouse models, which exhibit compromised cartilage architecture and heightened OA susceptibility with age [127,129,130]. Additionally, matrilin-3 contributes to the hydrophilic and viscoelastic properties of cartilage through its ability to bind and induce aggrecan—a critical proteoglycan responsible for shock absorption and tensile resilience in joint tissue [131,132]. While this function has not been directly studied in hand OA, these findings offer plausible mechanisms by which *MATN3* variants may contribute to localized cartilage weakening and joint degeneration in the thumb CMC joint.

## 7. Beyond the Influence of Genetic and Inflammatory Factors in Hand OA

### 7.1. Epigenetic Regulation of Hand OA Pathophysiology

Epigenetic changes, such as DNA methylation and histone modifications, have been shown to play a crucial role in the development of OA across various joints [40,42,43,44]. While some epigenetic changes are shared across multiple joint sites, recent studies have emphasized the importance of joint-specific epigenetic mechanisms, particularly in hand OA [133,134,135]. A genome-wide DNA methylation study analyzed over 1000 individuals with hand OA from the Framingham Offspring Cohort, revealing differentially methylated CpG sites (FDR < 0.05) associated with the disease [41]. One of the top CpG sites, cg12762517, was located in the *PARP3* gene, which encodes a poly-ADP-ribosyl transferase involved in DNA repair, regulation of apoptosis, and stress response [136,137,138]. This suggests that DNA damage and repair mechanisms may be involved in hand OA pathogenesis, with PARP3 playing a central role. No prior studies had associated this region with OA, making this a novel discovery in the epigenetic landscape of hand OA. Mechanistic studies into the role of PARPs in other diseases have shown PARPs as coactivators of the NF-κB signaling cascade [139,140,141]. Therefore, PARP3 could mediate its impact on hand OA by activating the NF-κB-induced inflammatory pathway.

The study also identified cg20312179, a CpG site associated with the expression of the *EPS15* gene, which was in turn linked to the expression of 40 other genes. These genes were enriched in pathways related to bone mineralization, osteoblast differentiation, and innate immunity, highlighting a potential connection between altered methylation patterns, bone metabolism, and immune responses in hand OA. For instance, EPS15 is known to mediate EGFR endocytosis, which, as previously discussed, is the receptor for TGFα [142]. TGFα has been implicated in both protective and destructive roles in chondrocytes.

Taken together, these findings suggest that differential methylation of CpG sites influences gene expression in hand OA, potentially altering key processes involved in cartilage homeostasis, immune response, and bone remodeling.

Another study evaluated the methylation status of several regulatory sites within genes linked to hand OA predisposition in a cohort of Finnish women, revealing significant epigenetic modifications associated with hand OA severity [73]. The *COL2A1* gene, which encodes type II collagen, an essential component of cartilage, was found to be epigenetically regulated through DNA methylation at specific CpG sites. Methylation at this site was significantly associated with radiographic hand OA affecting three or more joints (*p* = 0.04). This suggests that epigenetic modifications in *COL2A1* may influence the structural integrity of cartilage in hand OA by modulating collagen synthesis, a key determinant of cartilage homeostasis and degeneration. Additionally, methylation of the *ALDH1A2* gene—which plays a role in retinoic acid biosynthesis and has been linked to cartilage differentiation—was associated with the summary score of radiographic findings (*p* = 0.02). Stratification by occupation revealed that these associations were stronger in teachers than in dentists, suggesting possible occupational influences on methylation patterns. This highlights the complexity of gene–environment interactions in hand OA, with epigenetic regulation mediating genetic susceptibility and environmental factors, such as occupational hand use.

A more recent study identified elevated methylation levels in the *BMP7* gene, a key regulator of cartilage repair and chondrocyte differentiation, in patients with hand OA [135]. This epigenetic modification may impair the anabolic functions of BMP7, leading to accelerated cartilage degradation. Further studies are required to determine the utility of *BMP7* methylation in peripheral leukocytes as a minimally invasive systemic biomarker for the early diagnosis and progression of hand OA.

### 7.2. Emerging Gut Connection

Dysbiosis, or an imbalance in the gut microbiome, has been implicated in various inflammatory conditions, and emerging evidence suggests a similar connection in hand OA [143,144,145]. The Xiangya Osteoarthritis Study, a large population-based cohort study involving 1388 participants, investigated the relationship between the gut microbiome and symptomatic hand OA. The study found that beta-diversity, which measures the difference in microbial community composition between individuals, was significantly associated with symptomatic hand OA [146]. However, alpha diversity, which indicates the variety of species within an individual, was not significantly different between those with and without hand OA, suggesting that while the overall richness of the microbiome may not differ, the specific microbial compositions are distinct in those with symptomatic hand OA.

Further analysis showed that individuals with symptomatic hand OA had an increased relative abundance of the genera *Bilophila* and *Desulfovibrio*, both sulfate-reducing bacteria, and a lower relative abundance of *Roseburia*. *Bilophila* and *Desulfovibrio* have been implicated in other inflammatory diseases including inflammatory bowel disease, irritable bowel syndrome, and colorectal cancer [147,148,149,150,151].

**Figure 4:**
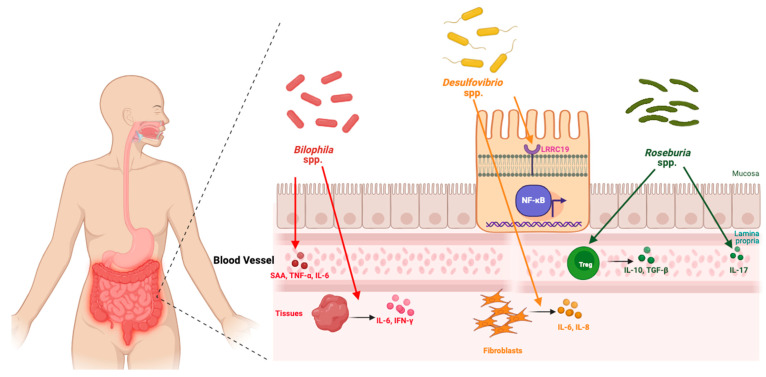
The gut–joint axis in hand OA. *Bilophila* spp. and *Desulfovibrio* spp. promote inflammation via cytokine induction and NF-κB activation, while *Roseburia* spp. counteract inflammation through Treg-mediated anti-inflammatory pathways.

Studies in animal models of obesity and metabolic dysregulation have shown that *Bilophila* induces the expression of inflammatory cytokines, IFN-γ and IL-6, in various tissues as well as LBP (lipopolysaccharide binding protein), A-SAA (SAA acute-phase reactant serum amyloid A), TNF-α, and IL-6 in serum (Figure 4) [148,149]. These findings suggest that *Bilophila* drives both local and systemic inflammation. Similarly, *Desulfovibrio* has been shown to induce the transcription of inflammatory genes and activate the NF-κB pathway via LRRC19 [150]. *Desulfovibrio* also stimulates the production of IL-6 and IL-8 in human oral epithelial and gingival fibroblast cells in response to its bacterial products, such as lipopolysaccharides [152,153,154]. In summary, these studies provide initial insights into the mechanisms by which *Bilophila* and *Desulfovibrio* may contribute to symptomatic hand OA, possibly through promoting inflammation (Figure 4). *Roseburia*, in contrast, is typically associated with anti-inflammatory effects [155,156]. Studies examining the role of *Roseburia* in other diseases demonstrate that it mitigates inflammation through various pathways (Figure 4). These include the inhibition of lipopolysaccharide (LPS)-induced IL-17 secretion and the promotion of regulatory T cells (Treg) cell differentiation and expression (Figure 4). Treg cells are known to suppress inflammation by producing anti-inflammatory cytokines such as IL-10 [157,158,159]. These pro-and anti-inflammatory effects demonstrate that the complex gut microbiome likely has a significant effect on fine-tuning the initiation and propagation of inflammation, thereby contributing to hand OA pathophysiology. Further research is necessary to clarify the precise role of these organisms in hand OA-specific models.

Functional pathway analysis in the Xiangya Osteoarthritis Study also indicated significant alterations in amino acid, carbohydrate, and lipid metabolic pathways in individuals with symptomatic hand OA suggesting that the gut microbiome could also significantly influence systemic metabolic states, contributing to the pathogenesis of hand OA. More recent evidence has also identified significant alterations in the tryptophan metabolism of the gut microbiota in patients with hand OA [146]. Participants in the discovery cohort with symptomatic hand OA had higher levels of plasma 5-hydroxyindoleacetic acid (5-HIAA) and 5-hydroxytryptophol (5-HTOL). The elevated levels of 5-HIAA and 5-HTOL metabolites in the serotonin pathway have been implicated in erosion and pain in hand OA [160]. Conversely, these participants had reduced levels of indole-3-lactic acid (ILA), skatole, and 3-hydroxyanthranilic acid (3-HAA), which are anti-inflammatory metabolites in the indole and kynurenine pathways. ILA, skatole, and 3-HA have all been implicated in quelling inflammation in various diseases [161,162,163,164]. In addition, ILA plays a critical role in maintaining gut barrier integrity and modulating systemic inflammation [165], suggesting that gut microbiome dysbiosis in hand OA patients skews tryptophan metabolism towards pro-inflammatory pathways and disrupts anti-inflammatory processes, consequently exacerbating hand OA symptoms and progression. Taken together, multiple studies have highlighted the intricate link between gut microbiota, metabolism, and host physiological processes in the hand OA joint.

This emerging gut–joint axis provides a novel perspective on the pathophysiology of hand OA and suggests potential therapeutic avenues to target the gut microbiome with the goal of mitigating inflammation and slowing hand OA progression. While modulating the gut microbiome could alleviate inflammation and improve joint health in OA patients, the largely important question of how we modulate the gut microbiome specifically to target a particular disease, hand OA in focus here, remains to be answered.

## 8. Randomized Controlled Trials (RCTs) in Hand OA

Given the vast number of therapeutic research studies in hand OA, this review focuses on randomized controlled trials (RCTs) published within the past 15 years that directly target the molecular and cellular pathways we have discussed in prior sections as implicated in hand OA pathogenesis. Specifically, we examined the existing literature for interventions modulating inflammatory cytokines (e.g., IL-1β, TNFα, IL-6), epigenetic regulation, cartilage metabolism, and other key signaling pathways discussed in the prior sections. By anchoring our analysis to these targets, this review critically evaluates whether insights derived from in vitro and in vivo studies translate into clinically meaningful symptom relief and functional improvement in patients with symptomatic hand OA.

### 8.1. Methods

To identify relevant studies, a comprehensive literature search was conducted across PubMed, Embase, and Cochrane Central Register of Controlled Trials (CENTRAL) using a combination of MeSH terms and keywords related to hand osteoarthritis (“hand OA”, “carpometacarpal OA”, “interphalangeal OA”), targeted molecular pathways (“inflammatory cytokines”, “IL-1β”, “TNFα”, “IL-6”, “WNT signaling”, “retinoic acid metabolism”, “adipokines”, “gut microbiome”, “Bilophila”, “Desulfovibrio”, “Roseburia”, “pro-inflammation”, “anti-inflammation”, “TEAD1”, “NR3C1”, “MGP”, “vitamin K”, “TGFα”), and intervention types (“intra-articular injections”, “biologics”, “prolotherapy”, “platelet-rich plasma”, “hyaluronic acid”, “corticosteroids”, “targeted therapies”, “microbiome-modulating interventions”, “adipokine-targeting therapies”). The search was limited to randomized controlled trials published in the past 15 years, from 2010 to 2025, and studies had to be available in English.

Studies were included if they met the following criteria: (1) randomized controlled trials evaluating pharmacological or biologic interventions that explicitly target molecular and cellular mechanisms implicated in primary hand OA—the non-erosive, non-autoimmune form of the disease; (2) studies assessing clinically meaningful outcomes such as pain relief, functional improvement, inflammatory biomarker modulation, or structural joint changes; and (3) trials that enrolled participants with confirmed radiographic or clinical diagnosis of hand OA, whether involving the carpometacarpal (CMC) joint or interphalangeal (IP) joints.

Exclusion criteria included (1) observational studies, case series, and non-randomized trials; (2) studies that focused on hand OA treatments without a clear mechanistic link to pathways discussed in this review; (3) trials assessing multimodal interventions where the effect of the pharmacologic agent could not be isolated; and (4) studies investigating systemic therapies without direct relevance to joint-specific inflammatory or cartilage-regenerative mechanisms in hand OA. Additionally, studies explicitly evaluating erosive hand OA or rheumatoid arthritis were excluded due to their distinct pathophysiology, which falls outside the scope of this review. The selection process followed PRISMA guidelines, with titles and abstracts screened for relevance before full-text review.

### 8.2. Methotrexate (MTX)

Methotrexate (MTX) is a disease-modifying antirheumatic drug (DMARD) widely used in rheumatoid arthritis (RA) and other autoimmune arthritides due to its potent immunosuppressive and anti-inflammatory effects [166,167]. Until recently, its interest in hand OA had been limited to erosive hand OA, where inflammatory mechanisms overlap with those seen in inflammatory arthritis [168]. MTX is known to increase extracellular adenosine levels, which downregulate key pro-inflammatory cytokines, including TNFα, IL-1β, and IL-6—all of which have been implicated in hand OA pathogenesis [169,170].

The METHODS trial was the first RCT to evaluate MTX in non-erosive hand OA with synovitis [171]. A total of 97 patients with symptomatic IP or thumb CMC OA (KL grade ≥ 2) and MRI-confirmed synovitis were randomized to receive oral MTX (15 mg/week) or placebo for six months, with assessments at 6 and 12 months. Patients in the MTX group experienced a significantly greater reduction in pain compared to placebo at 6 months, supporting a potential role for inflammation modulation in pain relief. While pain and stiffness improved, no significant differences were observed in functional measures such as grip strength, Functional Index for Hand Osteoarthritis (FIHOA), or Australian/Canadian Osteoarthritis Hand Index (AUSCAN) function scores. Pain reduction was not immediate, aligning with MTX’s known delayed onset, with notable improvements emerging after three months.

While the METHODS trial provides the first high-quality evidence evaluating MTX in non-erosive hand OA, future studies may benefit from larger patient populations and longer treatment durations to observe the long-term structural modification of the affected joint and the prolonged effects of MTX.

### 8.3. Botulinum Toxin A (BoNT-A)

Botulinum toxin A (BoNT-A) is a neurotoxin that inhibits acetylcholine release at the presynaptic terminal of the neuromuscular junction, resulting in temporary localized muscle paralysis [172]. Beyond its established use in movement disorders and spasticity, BoNT-A also exerts antinociceptive effects through inhibition of peripheral neuropeptides such as substance P, calcitonin gene-related peptide (CGRP), and glutamate [173]. These neuropeptides contribute to neurogenic inflammation, which plays a key role in synovitis and pain sensitization in OA [174,175]. In preclinical generalized OA models, intra-articular BoNT-A has been shown to reduce levels of pro-inflammatory cytokines including IL-1β and TNF-α in synovial tissues, alongside attenuated cartilage degradation and inflammatory cell infiltration [176].

A phase 3 study investigated the efficacy of intra-articular BoNT-A in patients with painful thumb CMC OA [177]. A total of 60 participants were randomized to receive a single intra-articular injection of BoNT-A (50 Allergan units in 1 mL saline) or a placebo injection of saline alone. At 3 months, BoNT-A recipients experienced significantly greater pain reduction compared to placebo, with improvements evident as early as 1 month and persisting through the 3-month mark. However, this benefit was not sustained at 6 months, suggesting a time-limited therapeutic effect. Functional outcomes, including grip strength and hand-specific functional assessments did not significantly differ between groups. Notably, BoNT-A was well tolerated, although 47% of patients reported mild and transient motor weakness in the thenar region, likely reflecting diffusion of the neurotoxin to adjacent motor units.

The RHIBOT trial provides the first high-quality evidence supporting BoNT-A as a short-term intra-articular analgesic in thumb CMC OA. Nonetheless, further research is needed to explore optimal dosing regimens, the potential utility of repeat injections, and whether BoNT-A can provide meaningful symptom relief in IP OA.

### 8.4. Topical Cetylated Fatty Acids (CFAs)

Cetylated fatty acids (CFAs) are a group of naturally occurring fats with reported anti-inflammatory and lubricating properties that have been investigated in various musculoskeletal conditions, including OA [178,179,180]. In vitro studies suggest that CFAs stimulate chondrogenesis while downregulating pro-inflammatory cytokines such as TNF-α and IL-6 [181]. While their efficacy has been explored in knee, elbow, and wrist OA, data on their effects in hand OA remain sparse [179,180].

The only randomized controlled trial to evaluate topical CFA therapy in hand OA was conducted in 72 patients with symptomatic CMC or IP involvement [178]. Participants were randomized to apply either CFA cream or placebo twice daily for 6 weeks. The primary outcome measure, the FIHOA, did not show a significant difference between the CFA and placebo groups. However, secondary outcomes revealed significant symptom relief in the CFA-treated group compared to placebo. At 6 weeks, patients receiving CFA cream reported significantly lower pain scores and better overall symptom improvement on the Patient Global Assessment (PGA).

These findings suggest that while CFA application did not enhance functional capacity, it provided modest pain relief and improved patients’ perceived disease burden. Although these results highlight CFAs as a potential symptom-relieving therapy for hand OA, future studies should explore longer treatment durations, head-to-head comparisons with established topical agents (e.g., NSAIDs, capsaicin), and potential synergistic effects with other therapies to better define the clinical utility of CFAs in hand OA management.

### 8.5. Tocilizumab: IL-6 Receptor (IL-6R) Blockade

Tocilizumab, a monoclonal antibody targeting the IL-6 receptor (IL-6R), has been explored as a treatment for hand OA based on mechanistic data implicating IL-6 in hand OA pathogenesis as previously discussed [182]. Preclinical studies in animal OA models show that IL-6 blockade reduces cartilage damage and alleviates mechanical allodynia, while observational data associated high IL-6 levels with increased OA severity and pain [183,184].

In a 12-week study, 91 patients with symptomatic hand OA involving DIP or PIP joints were randomized to receive intravenous tocilizumab (8 mg/kg, given twice four weeks apart) or placebo [182]. Change in hand pain at week 6 did not differ between the tocilizumab and placebo groups. Similarly, no significant differences were observed in any secondary outcomes, including swollen joint count, stiffness, FIHOA scores, or PGA. Subgroup analysis of patients with clinically swollen joints at baseline also showed no treatment effect.

These findings were unexpected given the established role of IL-6 in hand OA-related inflammation and joint degradation. Several factors may explain the negative results. First, while IL-6 contributes to structural progression in OA, its role in mediating pain may be more limited or indirect. Pain in hand OA is increasingly understood as multifactorial, involving not only synovitis but also bone marrow lesions, subchondral remodeling, and central sensitization, which may not be adequately modulated by IL-6 blockade [185,186,187]. Second, the level of inflammation in enrolled patients may have been insufficient to benefit from immunomodulatory therapy, particularly as synovitis was assessed clinically rather than through imaging. Third, the extent to which systemically administered tocilizumab reached therapeutic concentrations in the affected joints remains uncertain, potentially limiting its efficacy in localized disease. Finally, nociceptive drivers such as nerve growth factor (NGF), Toll-like receptor signaling, and chemokine pathways may predominate in advanced disease, rendering IL-6 inhibition inadequate. Although the results do not support the use of IL-6 inhibitors for pain relief in hand OA, they raise important questions about the disconnect between molecular inflammation and clinical symptoms in hand OA. Further studies are needed to assess whether IL-6 blockade may slow structural progression or better benefit other subtypes of hand OA with more overt inflammatory features.

### 8.6. Adalimumab: TNF-α Blockade

Adalimumab is a monoclonal antibody targeting TNF-α, a central inflammatory mediator implicated in cartilage degradation, synovial inflammation, and nociceptor sensitization in hand OA, as previously discussed in the pathogenesis section [34,35,36]. Despite strong biologic plausibility, most clinical trials of TNF-α inhibitors have focused on erosive and autoimmune hand OA subtypes, with only one RCT specifically evaluating TNF-α blockade in primary hand OA [188].

In this double-blind trial, 85 patients with symptomatic hand OA were randomized to receive either adalimumab 40 mg subcutaneously (administered twice, 15 days apart) or placebo, with follow-up over 26 weeks. The primary outcome—achievement of ≥50% reduction in hand pain on the visual analog scale (VAS) at week 6—did not differ significantly between groups. Secondary endpoints, including changes in joint counts, function, stiffness, and global assessments, also showed no treatment effect, with the exception of a modest but statistically significant reduction in swollen joint count in the adalimumab group at week 26. Biomarker analyses revealed no significant changes in serum cytokines or cartilage turnover markers (TNF-α, IL-6, CTX-II, COMP). Subgroup analysis of participants with ≥3 swollen joints at baseline also failed to demonstrate a differential response.

Several factors may account for the lack of efficacy: (1) the limited dosing regimen (only two injections), which may have been insufficient to achieve sustained TNF-α suppression; and (2) the possibility that TNF-α, while mechanistically implicated in OA pathogenesis, may not be the dominant driver of pain in established hand OA. Similarly to findings from the tocilizumab trial, these results raise important questions about the extent to which suppressing molecular inflammation translates into meaningful pain relief in symptomatic hand OA.

### 8.7. Colchicine

Colchicine, a microtubule polymerization inhibitor with anti-inflammatory properties, has been hypothesized to reduce symptoms in OA by attenuating inflammasome activation and lowering cytokine levels such as IL-1β and IL-6 [189]. While it is effective in treating crystal-induced arthritis and has shown potential in select knee OA studies, its utility in hand OA remains unclear [190,191,192].

Two RCTs have now evaluated colchicine in patients with symptomatic primary hand OA. The first trial, COLAH, enrolled 64 participants with symptomatic and painful hand OA, including both thumb CMC and IP OA [193]. From the cohort, 40% of patients were deemed via X-ray to have non-erosive primary OA. Patients received either 0.5 mg of colchicine twice daily or placebo for 12 weeks. Pain measures did not differ significantly between groups at week 12. No secondary outcomes, including grip strength, joint tenderness or swelling, C-reactive protein, or Michigan Hand Questionnaire scores, showed meaningful differences.

A second trial, COLOR, similarly failed to demonstrate efficacy [194]. A total of 100 participants with symptomatic hand OA and finger pain ≥40 mm were randomized to receive colchicine at a dose of 0.5 mg twice daily or placebo for 12 weeks. The primary outcome—change in target hand finger pain on a 100 mm VAS—was identical between groups. No significant between-group differences were observed in global assessments, AUSCAN pain and function scores, grip strength, or tender joint counts.

The negative findings in both trials do not support colchicine as an effective symptomatic therapy for hand OA. Despite biologic plausibility and prior encouraging data from knee OA studies, colchicine appears to be ineffective in hand OA.

### 8.8. Corticosteroids

Corticosteroids are anti-inflammatory drugs commonly used in the management of symptomatic OA [195,196,197]. Their appeal in hand OA stems from their ability to suppress key inflammatory mediators implicated in OA pathogenesis—such as IL-1β, IL-6, and TNF-α—which contribute to pain sensitization, synovitis, and cartilage degradation [198,199,200,201].

Oral corticosteroids have been evaluated in two RCTs with contrasting results. The HOPE study assessed oral prednisolone (10 mg/day for 6 weeks followed by a 2-week taper) in 92 patients with symptomatic hand OA and clinical signs of synovitis [202]. Compared to placebo, the prednisolone group experienced significantly greater reductions in finger joint pain, as well as improvements in hand function and ultrasound-detected synovial inflammation. In contrast, another trial evaluating low-dose prednisolone (5 mg/day for 4 weeks) in 70 patients with hand OA found no significant difference from placebo in pain scores, hand function, or joint counts, highlighting a potential dose–response relationship or need for greater baseline inflammation to observe benefit [203].

Intra-articular corticosteroids have also produced variable outcomes depending on the target joint. One trial evaluated intra-articular triamcinolone hexacetonide (5 mg) versus saline in 40 patients with thumb CMC OA [204]. At 24 weeks, there was no significant difference between groups in pain reduction, stiffness, or global assessments. The lack of benefit in this study may reflect limited intra-articular space in the CMC joint or resistance of more advanced disease to anti-inflammatory effects. Conversely, another trial examined intra-articular triamcinolone hexacetonide (20 mg/mL) with 2% lidocaine versus lidocaine alone in 60 patients with IP OA and found significantly greater improvements in pain on movement and joint swelling through week 12 in the corticosteroid group [23]. However, no differences were observed in grip strength, pinch strength, or pain at rest, suggesting that functional gains remain limited even when symptomatic improvement occurs.

Topical corticosteroids have also been tested. One RCT evaluated topical betamethasone dipropionate (0.5 mg/g ointment) applied three times daily for six weeks in 106 patients with symptomatic hand OA [205]. No significant differences were observed between corticosteroid and placebo groups in pain reduction, function, or stiffness, though the treatment was safe and well tolerated. The lack of efficacy may be attributable to inadequate transdermal penetration and limited joint-specific drug delivery.

Together, these studies reveal a nuanced and context-dependent picture of corticosteroid efficacy in hand OA. Oral corticosteroids may offer short-term relief in patients with clinically evident synovitis, but lower doses and non-targeted patient populations yield mixed results. Intra-articular injections appear more effective for IP joints than for the thumb CMC joint, while topical applications have not shown meaningful benefit. These findings highlight the importance of tailoring corticosteroid treatment to joint type, disease activity, and delivery route in hand OA management.

### 8.9. Platelet-Rich Plasma (PRP)

Platelet-rich plasma (PRP) is an autologous blood-derived preparation enriched with platelets, growth factors, and bioactive molecules known to support tissue healing, modulation of inflammation, and regeneration [206,207]. PRP has garnered substantial interest in the management of OA, including hand OA, due to its potential for regenerative effects and its capacity to modulate key inflammatory pathways involved in OA pathogenesis via the receptor antagonist for interleukin-1 (IL-1ra) and other soluble receptors targeting TNF (sTNF-R), IL-4, and IL-10 receptors [208]. Despite this biological rationale, clinical evidence specifically evaluating PRP in hand OA remains sparse. To date, only two RCTs have evaluated PRP efficacy, all exclusively in thumb CMC OA, leaving significant uncertainty regarding its efficacy in IP OA.

In a blinded trial, intra-articular injections of autologous fat, PRP, the combination of autologous fat and PRP, or placebo (0.9% saline) were evaluated in 95 patients with thumb CMC OA [209]. At a mean follow-up of two years, only the combination of fat and PRP resulted in significant and sustained pain reduction compared with placebo. Additionally, this combination demonstrated clinically relevant improvements in hand function and overall quality of life. In contrast, injections of PRP alone showed inferior pain reduction compared to placebo, highlighting the importance of combining autologous substances to achieve clinically relevant long-term benefits.

Another trial examined the use of autologous conditioned plasma combined with stromal vascular fraction (SVF) and adipose-derived stem cells in 30 hands with thumb CMC OA [210]. Patients received intra-articular injections of this composite biologic, with outcomes assessed at 6 and 24 months. The treatment resulted in statistically significant reductions in pain and improved functional outcomes, especially in stages 2 and 3 of the disease. The composite biologic technique showed promise in potentially delaying the need for resection arthroplasty.

The only other RCT evaluating PRP in hand OA compared intra-articular PRP with corticosteroids (methylprednisolone with lidocaine) in 33 patients with symptomatic thumb CMC OA [211]. This trial demonstrated superior pain relief and improved functionality with PRP compared to corticosteroids at the 12-month follow-up, suggesting PRP may offer sustained clinical advantages.

Although these studies indicate strong clinical evidence for PRP in managing thumb CMC OA, the absence of RCTs addressing IP joint OA significantly limits the generalizability of these findings across the diverse spectrum of hand OA presentations.

### 8.10. Hypertonic Dextrose Therapy (Prolotherapy)

Hypertonic dextrose therapy (prolotherapy) is an injection-based therapy involving the administration of hypertonic dextrose solution into or around joints and soft tissues, with the goal of stimulating a localized inflammatory response that induces tissue repair through the upregulation of growth factors such as transforming growth factor-beta (TGF-β), insulin-like growth factor-1 (IGF-1), and platelet-derived growth factor (PDGF) [212,213,214]. These growth factors are hypothesized to enhance cartilage regeneration and provide structural joint support, directly addressing key pathological mechanisms previously discussed in hand OA, such as chronic inflammation and impaired cartilage repair [215].

An RCT compared prolotherapy with paraffin wax therapy in 42 patients with bilateral hand OA, including symptomatic CMC, DIP, and PIP joints. Both treatments significantly reduced pain and improved grip strength at 3 months; however, prolotherapy resulted in significantly greater improvements in overall hand function [216]. While these results suggest potential clinical benefits of prolotherapy, the absence of subgroup analyses by specific joint type limits the ability to discern differential efficacy across distinct joints involved in hand OA.

Despite encouraging initial results from this study, significant limitations remain, including small sample size, limited follow-up duration, and lack of subgroup characterization of joint involvement. Future studies should emphasize standardized treatment protocols, precise joint characterization, and robust long-term outcomes to fully determine the clinical utility and therapeutic potential of prolotherapy in managing hand OA.

### 8.11. Estrogen Replacement Therapy

Estradiol (E2) at physiologic levels has been shown to downregulate pro-inflammatory cytokines such as IL-6 and TNF-α while promoting anti-inflammatory mediators like IL-10 and TGF-β [217]. The postmenopausal decline in E2 levels is associated with a shift toward a more pro-inflammatory immune environment and enhanced nociceptive sensitization, raising the possibility that estrogen therapy may offer therapeutic benefit in postmenopausal hand OA [217,218].

The HOPE-e trial was a feasibility RCT designed to assess the acceptability and safety of conjugated estrogens plus bazedoxifene (a selective estrogen receptor modulator) in postmenopausal women with symptomatic hand OA [219]. A total of 73 participants were randomized to receive either hormone therapy or placebo for 24 weeks. While both groups experienced reductions in hand pain over the treatment period, there was no statistically significant difference between groups in either daily or recalled pain measures. No effects were observed on grip strength or hand function.

Interestingly, following tapering and discontinuation of the drug, nearly half (46%) of participants in the hormone therapy group reported worsening hand pain, compared to only 17% in the placebo group—suggesting a possible withdrawal effect or delayed symptom recurrence. Additionally, quality of life and menopause-related symptoms improved in the treatment group, consistent with established hormone therapy benefits.

Although not designed to determine treatment efficacy, the HOPE-e trial provides preliminary support for the feasibility of larger trials. Future studies with longer treatment durations, adequately powered sample sizes, and enrichment for hormone-sensitive OA phenotypes (e.g., abrupt estrogen withdrawal) are warranted to determine whether estrogen-based therapy can modify symptoms or disease trajectory in postmenopausal hand OA.

### 8.12. Hydroxychloroquine (HCQ)

Hydroxychloroquine (HCQ), a disease-modifying antirheumatic drug commonly used in inflammatory arthritides such as rheumatoid arthritis, has been investigated in hand OA due to its immunomodulatory properties, including inhibition of Toll-like receptor signaling and downregulation of IL-1β, IL-6, and TNFα production [220,221]. However, results from two randomized controlled trials do not support its use for symptomatic relief in primary hand OA.

A 24-week study was conducted with 196 patients with primary hand OA who were randomized to receive either HCQ 400 mg daily or placebo [222]. The primary outcome—change in hand pain—showed no significant difference between groups. Secondary outcomes, including stiffness, function, and quality of life across physical, emotional, and social domains, were also unchanged. No significant subgroup effects were seen, including among patients with higher baseline pain levels or radiographic severity.

A second larger trial further reinforced these findings. The HERO RCT was a 12-month study involving 248 patients with symptomatic hand OA who received either HCQ (200–400 mg/day) or placebo [223]. The primary endpoint—hand pain—showed no significant difference. Secondary endpoints, including AUSCAN pain and function, grip strength, radiographic progression, and ultrasound synovitis scores, also revealed no treatment effect.

Together, these two high-quality studies demonstrate that HCQ does not offer symptom-modifying benefit for hand OA and should not be routinely prescribed for this indication.

### 8.13. Hyaluronic Acid (HA)

Hyaluronic acid (HA), also known as hyaluronan, is a naturally occurring glycosaminoglycan abundantly present in synovial fluid, where it plays crucial roles in joint lubrication, shock absorption, and maintaining cartilage integrity [224,225]. In OA, the concentration and molecular weight of HA in synovial fluid are often reduced, leading to decreased viscosity and elasticity, which impairs joint function and contributes to cartilage degradation [226,227]. Therapeutically, intra-articular injections of HA aim to restore the viscoelastic properties of synovial fluid, thereby improving joint lubrication and cushioning [228]. Additionally, HA has been shown to exhibit anti-inflammatory and chondroprotective effects by inhibiting pro-inflammatory cytokines such as IL-1β and TNFα [229,230].

A double-blind, multicenter trial evaluated Hylan G-F 20 compared to triamcinolone acetonide and bupivacaine injections in 200 patients with thumb CMC OA [231]. At 26 weeks, all treatment arms showed significant pain improvement, but no significant differences were observed between groups, indicating that Hylan G-F 20 was not superior to corticosteroids or local anesthetic injections.

Another study compared intra-articular HA to extracorporeal shock wave therapy (ESWT) in 58 patients with thumb CMC OA [232]. Both groups demonstrated significant improvements in pain and hand function at 6 months; however, patients receiving ESWT experienced greater pain relief and improved pinch strength than those treated with HA injections. These findings suggest that while HA injections offer meaningful clinical improvement, their effect may be less pronounced than other non-invasive modalities.

### 8.14. Efficacy of Corticosteroids vs. Other Intra-Articular Therapies

Interest has grown in exploring alternatives to corticosteroids such as HA, PRP, and prolotherapy that may offer regenerative potential or more durable pain relief with fewer safety concerns than those associated with repeated corticosteroid use. Multiple randomized trials have compared these alternatives head to head with corticosteroids, particularly in thumb CMC OA.

In a three-arm study involving 45 patients, intra-articular PRP, HA, and corticosteroids were evaluated for efficacy [233]. All groups experienced improvement in pain and function at 4 weeks, but by 12 weeks, only the HA group maintained its benefits, while the corticosteroid and PRP groups showed clinical deterioration. These findings suggested that although corticosteroids provide rapid relief, HA may sustain improvements for longer.

Another trial evaluated HA versus betamethasone in 88 patients with KL grade II–III thumb CMC OA [234]. While overall group comparisons showed no significant differences in outcomes, subgroup analyses revealed that HA provided greater functional gains in patients with more severe baseline pain and disability at 3- and 6-month follow-ups.

A third study involving 33 patients with Eaton–Littler grade I–III thumb CMC OA demonstrated that both PRP and corticosteroids improved symptoms at 3 months [211]. However, at 12 months, patients treated with PRP had significantly better outcomes in pain reduction, hand function, and satisfaction, underscoring PRP’s potential as a longer-lasting therapeutic option.

Prolotherapy has also emerged as a potential corticosteroid alternative. In a study of 60 patients with symptomatic thumb CMC OA, corticosteroid injections produced superior pain relief at 1 month, but by 6 months, the prolotherapy group demonstrated significantly greater pain reduction and functional improvement [235].

These trial-level insights are further contextualized by a recent network meta-analysis study comparing the efficacy of intra-articular corticosteroids, HA, PRP, and placebo in thumb CMC OA [236]. While no injection was superior to placebo in short-term analyses, PRP outperformed corticosteroids and placebo at medium-term follow-up in sensitivity analyses.

Taken together, these findings suggest that while corticosteroids remain effective for rapid symptom relief in thumb base OA, their benefit is often transient and less durable than emerging biologic and regenerative therapies. Intra-articular HA and PRP, though variable in efficacy, show promise for longer-term symptom control, particularly in selected patient subgroups. Prolotherapy may also represent a viable alternative with delayed but sustained benefits. However, the lack of consistent superiority, small sample sizes, and predominant focus on thumb CMC OA (with limited comparative data in IP joints) limit generalizability. Head-to-head trials with standardized endpoints and longer follow-up are needed to clarify whether these alternatives can truly surpass corticosteroids as the cornerstone of intra-articular therapy in hand OA.

### 8.15. Why Have Pharmacological Targeted Therapies Largely Failed to Deliver?

Despite considerable progress in understanding the molecular pathogenesis of hand OA—including the roles of pro-inflammatory cytokines such as TNF-α, IL-1β, and IL-6—no targeted therapy has yet demonstrated robust and sustained clinical benefit across broad patient populations. Several factors likely contribute to this translational gap. First, hand OA is a clinically and biologically heterogeneous disease, with significant variation in joint involvement, inflammatory burden, and patient-reported symptoms. This heterogeneity complicates trial design and dilutes therapeutic signal in unstratified populations. Second, while cytokines are mechanistically implicated in joint tissue remodeling and inflammation, their role as primary drivers of pain may be limited or context-dependent. Pain in hand OA is multifactorial, involving structural pathology, synovitis, subchondral bone changes, and neurogenic sensitization—all of which may not be modulated effectively by single-pathway interventions. Third, systemic delivery of cytokine-targeting biologics may not achieve adequate drug concentrations in small hand joints, particularly in the absence of florid synovitis.

It is important to note that some interventions—such as corticosteroids in IP OA, PRP in thumb OA, and methotrexate in patients with MRI-confirmed synovitis—have shown modest efficacy. However, these findings have not been sufficiently reproducible or generalizable to establish new standards of care. Furthermore, promising agents often demonstrate only short-lived benefit or are limited by logistical, safety, or regulatory barriers. Finally, therapeutic development in hand OA has been constrained by limited funding and underrepresentation in broader OA pipelines. Together, these challenges demonstrate the need for better disease phenotyping, multimodal interventions that target more than one mechanistic pathway in treating pain and locally delivered interventions that can be sure to achieve the necessary therapeutic concentrations in hand joints.

## 9. Conclusions

In summary, this review has examined recent advances in the molecular and genetic underpinnings of hand OA. Table 1 summarizes the key molecular mechanisms currently implicated in the pathogenesis of hand OA. While an interplay between genetic predisposition and inflammatory pathways is evident across multiple OA joints, hand OA is increasingly recognized as biologically distinct from large-joint OA, such as that of the hip and knee. In addition to classic inflammatory cytokines, key signaling cascades, such as TGFa and WNT, are also influenced by genetic predisposition. GWASs have identified hand-specific loci implicating inflammatory cytokines, chondrogenic pathways, and hormonal regulators, while emerging data on epigenetic modifications and gut microbiome interactions point to a broader systemic network of disease contributors. Whether this novel “gut–joint axis” operates differently in weight-bearing versus non-weight-bearing joints remains an open question.

In parallel, this review has also evaluated pharmacologic therapies tested in RCTs that have sought to target these molecular mechanisms. However, as reviewed in detail, many biologically plausible interventions—such as TNF and IL-6 inhibitors, hydroxychloroquine, colchicine, and corticosteroids—have shown limited or inconsistent efficacy in patient populations. These trial-level outcomes—mapped against their mechanistic targets—are summarized in Table 2. Emerging therapies such as MTX in synovitis-positive OA, PRP in thumb CMC OA, and prolotherapy in IP OA have shown early promise but require further validation in adequately powered, phenotypically enriched trials. Additionally, agents such as estrogen therapy and cetylated fatty acid creams highlight the importance of identifying patient subgroups (e.g., postmenopausal women) who may uniquely benefit from targeted interventions. The disconnect between molecular pathogenesis and treatment efficacy in hand OA punctuate the need for a new paradigm in hand OA therapeutic development—one that prioritizes longitudinal, biomarker-driven studies along with combination therapies that better address the multifactorial nature of pain and structural progression in hand OA. Only through this integrative approach can we hope to translate our understanding of hand OA pathogenesis into effective clinical therapies.

## Figures and Tables

**Table 1:** Summary of molecular mechanisms implicated in hand OA. This table summarizes key molecular pathways, mechanisms, and associated gene variants implicated in the pathogenesis of hand osteoarthritis (OA). References reflect genetic, preclinical, and translational human studies. Joint involvement is noted as thumb carpometacarpal (CMC), interphalangeal (IP), distal interphalangeal (DIP), proximal interphalangeal (PIP), or generalized hand OA when specific joints are not explicitly defined.

Mechanism Category	Molecular Factor	Role in Hand OA	Joint Involvement	Key References
**Cytokines**	TNFα	Pro-inflammatory; linked with cartilage breakdown and synovial inflammation	Hand OA	[37,45]
	IL-1β	Induces cartilage degradation; enhances inflammation via MMPs and ADAMTS	DIP OA, Hand OA	[47,50]
	IL-4	Inhibits IL-1β-induced protein expression of MMP-13; prevents ECM breakdown	Hand OA	[58]
	IL-6	Synovial inflammation; cartilage damage	Hand OA	[61,62]
	IL-7	Activates inflammatory T-cell responses; biomarker potential	Thumb CMC OA	[109]
**Other Signalling Pathways**	WNT9A	Regulates chondrogenesis; influences thumb CMC OA severity	Thumb CMC OA	[115]
	TGFα	Responds to mechanical stress; dual roles in cartilage catabolism/anabolism	Hand OA	[31]
	YAP/TAZ-TEAD	Mediates cartilage homeostasis; cellular response to mechanical cues	Hand OA	[31]
	ALDH1A2	Retinoic acid metabolism; impacts cartilage repair and homeostasis	Hand OA	[118,122]
**Epigenetic Factors**	DNA methylation of *PARP3*	DNA methylation affects DNA repair; modulates NF-κB signaling; influences inflammation and cartilage homeostasis	Hand OA	[41]
	DNA methylation of *EPS15*	Affects EGFR endocytosis and bone mineralization pathways; influences inflammation, immune response, and cartilage metabolism	Hand OA	[41]
	DNA methylation of *COL2A1*	Alters collagen type II synthesis, cartilage structural integrity; methylation associated with radiographic severity	Hand OA	[73]
	DNA methylation of *BMP7*	Impairs cartilage repair; elevated methylation linked to OA severity; potential systemic biomarker for hand OA diagnosis and monitoring	Hand OA	[135]
	Adiponectin	Lower levels linked with radiographic hand OA progression; potential protective role in OA through anti-inflammatory effects	Hand OA	[65]
	Resistin	SNPs associated with OA risk and severity; interacts with TNFα and IL-6 to exacerbate inflammation and OA progression	Hand OA	[66]
	Chemerin	SNPs associated with OA risk and pain severity; promotes inflammation via TLR4 and MAPK/ERK pathways	Hand OA	[66]
**Gut-Joint Axis**	*Bilophila*	Elevated in symptomatic hand OA; induces pro-inflammatory cytokines IFN-γ, IL-6, TNF-α, and systemic inflammatory markers; linked to inflammation in other inflammatory diseases	Hand OA	[146]
	*Desulfovibrio*	Elevated in symptomatic hand OA; stimulates inflammatory genes, activates NF-κB pathway, and produces IL-6 and IL-8; contributes to inflammation	Hand OA	[146]
	*Roseburia*	Reduced abundance in hand OA; typically anti-inflammatory; inhibits IL-17 secretion; promotes Treg cells and IL-10 production	Hand OA	[146]
**Extracellular Matrix Proteins**	MATN3	Chondrocyte metabolism; regulates the synthesis of ECM proteins	Thumb CMC OA	[123,124,125]
	MGP	Vitamin K-dependent; prevents cartilage calcification	Hand OA	[80,82,83]
**Glucocorticoid Receptor**	NR3C1	Modulates inflammation; regulates glucocorticoid response; influences cartilage and synovial homeostasis	Hand OA	[31]

**Table 2:** Summary of randomized controlled trials (RCTs) evaluating pharmacologic therapies in hand OA. This table summarizes randomized controlled trials investigating pharmacologic treatments for hand osteoarthritis (OA), including therapeutic mechanisms, joint-specific involvement, key clinical outcomes, and supporting references. Joint classification includes thumb carpometacarpal (CMC), interphalangeal (IP), distal interphalangeal (DIP), proximal interphalangeal (PIP), or generalized hand OA, as specified in each study.

Therapy	Mechanistic Target/Action	Joints Studied	Key Outcomes	References
**Methotrexate**	Reduction of inflammatory cytokines (TNFα, IL-1β, IL-6)	Thumb CMC, IP OA	Reduced pain at 6 months; no significant functional improvement	[171]
**Botulinum Toxin A**	Inhibition of neurogenic inflammation via neuropeptides; reduction of inflammatory cytokines (TNFα, IL-1β)	Thumb CMC OA	Short-term pain relief (3 months); transient motor weakness	[177]
**Topical Cetylated Fatty Acids (CFAs)**	Reduction of inflammatory cytokines (TNFα, IL-6)	Thumb CMC, IP OA	Modest pain relief; improved patient symptom perception	[178]
**Tocilizumab (IL-6 blockade)**	IL-6 receptor blockade; inflammation modulation	DIP, PIP OA	No significant clinical benefit compared to placebo	[182]
**Adalimumab (TNF-α blockade)**	TNF-α inhibition; inflammation modulation	Hand OA	No significant clinical benefit compared to placebo	[188]
**Colchicine**	Inflammasome inhibition; reduction of inflammatory cytokines (IL-1β, IL-6)	Thumb CMC, IP OA	No significant improvement over placebo	[193,194]
**Oral Corticosteroids**	Anti-inflammatory effects (IL-1β, IL-6, TNF-α inhibition)	Thumb CMC, IP OA	Short-term pain relief; mixed evidence on hand function	[202,203]
**Intra-articular Corticosteroids**	Anti-inflammatory effects (IL-1β, IL-6, TNF-α inhibition)	Thumb CMC, IP OA	Effective in pain relief for IP OA; limited effectiveness in thumb CMC OA	[23,204]
**Topical Corticosteroids**	Anti-inflammatory effects (IL-1β, IL-6, TNF-α inhibition)	Hand OA	No significant pain reduction compared to placebo	[205]
**Platelet-rich Plasma (PRP)**	Regenerative; reduction of inflammatory cytokines (IL-1β, TNFα); promotion of anti-inflammatory cytokines (IL-4, IL-10)	Thumb CMC OA	Improved pain and function, sustained in most trials	[209,210,211]
**Hypertonic Dextrose (Prolotherapy)**	Tissue regeneration via induced growth factors (TGF-β, IGF-1, PDGF)	Thumb CMC, IP, DIP, PIP OA	Improved function and pain vs. placebo	[216]
**Estrogen Replacement Therapy**	Anti-inflammatory effects (IL-6, TNF-α inhibition); promotion of anti-inflammatory cytokines (TGF-β, IL-10)	Hand OA	No significant improvement in hand pain vs. placebo	[219]
**Hydroxychloroquine (HCQ)**	Anti-inflammatory effects (TLR, IL-1β, IL-6, TNF-α inhibition)	Hand OA	No clinical benefit compared to placebo	[222,223]
**Hyaluronic Acid (HA)**	Joint lubrication; anti-inflammatory effects (IL-1β, TNF-α inhibition)	Thumb CMC OA	Comparable effectiveness to corticosteroids; moderate effects in hand pain and function	[231,232]

## Data Availability

No new data were created or analyzed in this study. Data sharing is not applicable to this article.
